# Clinical profile and Predictors of Outcomes of Hospitalized Patients with Laboratory-Confirmed Severe Acute Respiratory Syndrome Coronavirus 2 in Nigeria: A Retrospective Analysis of 13 High Burden States in Nigeria

**Published:** 2023-05-11

**Authors:** Christopher Sabo Yilgwan, Adamu Onu, Joshua Ofoli, Longji Benle Dakum, Nathan Yakubu Shehu, Dimie Ogoina, Ijeoma Okoli, Deborah Osisanwo, Vivian Okafor, Adebola Olayinka, Ibrahim Mamadu, Adebimpe Adebiyi

**Affiliations:** 1 Department of Paediatrics, University of Jos, Jos, Nigeria; 2 West African Center for Emerging Infectious Diseases, Jos, Nigeria.; 3 Nisa Garki Hospital, Abuja, Nigeria.; 4 Department of Internal Medicine, Jos University Teaching Hospital, Jos, Nigeria, Alex Ekwueme Way, Jabi, Abuja, Nigeria.; 5 Nisa Premier Hospital, Alex Ekwueme way, Jabi, Abuja, Nigeria; 6 Department of Medicine, Niger Delta University, Bayelsa, Nigeria.; 7 Department of Hospital Services, Federal Ministry of Health, Abuja, Nigeria.; 8 Ministry of Health, Abeokuta, Ogun State, Nigeria.; 9 Department of Medical Microbiology, Ahmadu Bello University, Zaria, Nigeria.; 10 2 Casablanca Street, off Aminu kano crescent, Wuse 2, FCT, Abuja, Nigeria.

**Keywords:** Clinical Profile, Predictors of Outcomes, Hospitalized Patients, SARS-CoV-2, COVID-19, High Burden States, Nigeria

## Abstract

**Background:**

The majority of global COVID deaths have occurred in developed countries. Not much is known about the clinical outcomes for the patients admitted with COVID in Nigeria. We thus described the clinical characteristics, outcomes, and predictors of outcomes of hospitalized Nigerian COVID-19 patients.

**Methodology:**

We performed multilevel and mixed effects regression, Kaplan-Meir survival, and Cox proportionate hazards analyses to evaluate factors associated with death in patients admitted for COVID-19 in 13 high-burden states of Nigeria between 25^th^ February 2020 and 30^th^ August 2021.

**Results:**

Of the 3462 patients (median age, 40 years (interquartile range 28 years 54 years), 2,990(60.6%) were male and, 213(6.15%) of them died while on admission. Male sex (adjusted odds ratio [aOR], 1.78 [95% confidence interval {CI}, 1.23–2.56]), age group 45–65 years (OR, 3.93 [95% CI, 1.29–12.02]), age group 66–75 years (aOR, 5.37 [95% CI, 1.68–17.14]), age group > 75 years (aOR, 6.81 [95% CI, 2.04–22.82]), chronic cardiac disease (aOR, 3.07 [95% CI, 1.20–7.86]), being diabetic (aOR, 2.16 [95% CI, 1.41–3.31]), and having chronic kidney disease (OR, 11.01 [95% CI, 2.74–44.24]),were strongly associated with increased odds of death. Having concurrent malaria (aOR, 0.45 [95% CI, 0.16–1.28]), use of Azithromycin for treatment (aOR, 0.33 [95% CI, 0.19–0.54]), and use of Chloroquine/Hydroxychloroquine for treatment (aOR, 0.07 [95% CI, 0.03–0.14]) were significantly associated with decreased odds of death.

**Conclusions:**

The cumulative probability of death of male patients, diabetics, hypertensives, and patients with CKD was higher than that of female patients and those without those comorbidities while concurrent malaria and use of chloroquine/hydroxychloroquine in the treatment regimen were associated with a decreased risk of dying in patients treated in our isolation centers.

## Introduction

The global COVID 19 pandemic has resulted in a high number of deaths and associated disruption of both public health and socioeconomic activities of countries and populations.[1,2] Majority of the global COVID deaths reported have occurred in developed and industrialized countries where the demography is quite different from Nigeria.2 Not much is known about the clinical outcomes for the patients admitted with COVID in Nigeria.[3] As at 20^th^ of August 2021, Nigeria had recorded 163,581 confirmed cases, with 150,005 cases discharged, 7,518 cases on admission, and 2,058 deaths with a significant increase in the number of confirmed cases and concurrent deaths since the beginning of the second and third waves of infections.[4]

Most of the reported COVID deaths in Nigeria have occurred in hospitals.[3] In addition, not much is known about the factors associated with these high in-hospital deaths underscoring the need for evaluating them.[3,4] Understanding the relative contributions and probable mechanisms for in-patient mortality in Nigeria could help us identify weak points within the health system that can be improved upon for current and future responses.[5] In addition, such a review will also help us plan and prioritize health infrastructure and resource allocation for better health outcomes in our public health system.[5]

We, therefore, set out to describe clinical characteristics and factors associated with the outcomes for patients hospitalized with laboratory-confirmed severe acute respiratory syndrome coronavirus 2 (SARS-CoV-2) infection early in Nigeria. In addition, we evaluated the predictors of mortality in those patients hospitalized with laboratory-confirmed severe acute respiratory syndrome coronavirus 2 (SRAS-COV-2) infection in Nigeria.

## Methodology

### Study design

This was a retrospective descriptive analysis of 3,462 patients with laboratory-confirmed SARS-CoV-2 infection seen and managed in the COVID-19 treatment centers across 13 high-burden states (Abia, Adamawa, Delta, Ebonyi, Kebbi, Kwara, Lagos, Niger, Ogun, Ondo, Osun, Oyo, Plateau, Taraba) in Nigeria and the Federal Capital Territory (FCT).

### Study Setting

This study utilized clinical records of patients seen in the COVID-19 treatment centers across the 13 high-burden states in Nigeria (Abia, Adamawa, Delta, Ebonyi, Kebbi, Kwara, Lagos, Niger, Ogun, Ondo, Osun, Oyo, Plateau, Taraba) and the federal capital territory (FCT). The COVID-19 treatment centers are located within the main tertiary care facilities in the states. These tertiary care facilities are either University Teaching Hospitals or Federal Medical Centers built for the purpose of research and training of specialist health manpower for the country. In addition, these treatment centers are mainly referral centers designated and equipped by the Government to cater to patients with COVID-19 in each of those states. All the staff manning the center were carefully selected, trained, and equipped to provide basic, specialist, and emergency care to patients admitted with COVID-19. In addition, each center had a site coordinator who was responsible for the day-to-day administration of the center as well as reporting to the head of the hospital. Standard operating procedures (SOP) for treatment, diagnosis, and management of COVID-19 developed by the WHO and adapted or adopted by the Nigerian Center for Disease Control (NCDC) were uniformly used in each treatment center. All staff were trained on the use and application of the SOP by a dedicated team of trainers from the NCDC and federal ministry of health (FMOH).

### Study population

We conducted a retrospective descriptive study of all patients admitted with real-time polymerase chain reaction (RT-PCR)–positive SARS-CoV-2 confirmed diagnosis across the treatment centers in Nigeria between 1^st^ April 2020 and 1^st^ August 2021. Those patients who were not admitted as well as those who had a negative test result even though treated for COVID-19 based on symptom scores were excluded from the review. Where a patient had 2 or more RT-PCR positive results, we included the first episode that required hospitalization.

### Sample size and Power calculation

**Figure 1:**
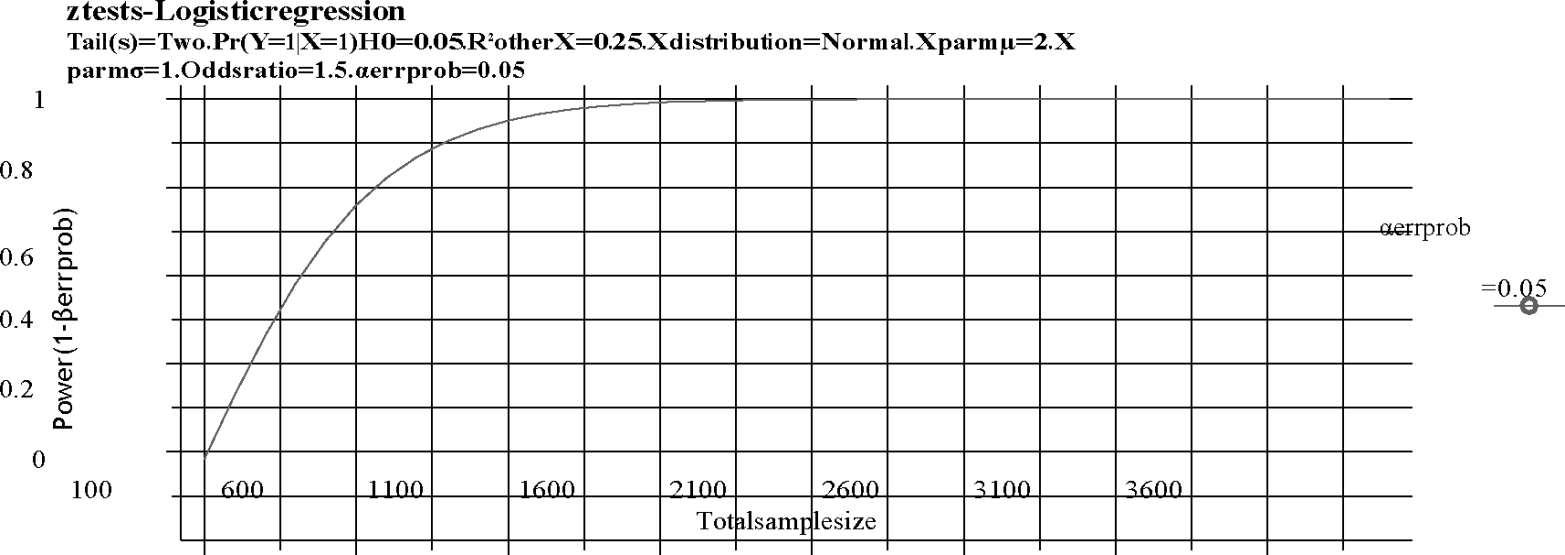
Plot of sample sizes versus power

The entire data set contained within the data management system for the 13 States was abstracted and used for the mortality review. The main explanatory covariate was the severity score assumed to be normally distributed based on the central limit theorem. Thus, using the G*Power statistical software,6 we determined that the number of subjects of 3,462 abstracted from the 13 high burden states had greater than 95% power for a two-tailed hypothesis test based on an *α*= 0.05, *β*= 1.5, *p*= 0.05, *R*^2^= 0.25 and assumed mean severity score of 2 with a standard deviation of 1 (see Figure 1).

### Ethical Considerations

We secured ethical approval from the National Health Research Ethics Committee (NHREC) and WHO AFRO ethics committee prior to the commencement of the mortality review to allow the for publishing of the key findings from the mortality review. Only variables needed for the review were abstracted and used. Patient identification and other identifiers linked to the patient were coded and anonymized in order to maintain patient confidentiality. The therapeutic option used in the treatment centers at the time was under emergency use trial and existing theoretical basis of CQ/HCQ as an Immunomodulator preceding WHO living guidance on therapeutics.

### Data Collection

We extracted data from the World Health Organization (WHO) nCOV database platform (Open Clinica Electronic Data Capture [EDC] System) for 28 sites across 15 states in Nigeria (see Figure 1). We retrieved data on the date of admission, enrolment date, demographic data (patients’ sex, age, health worker), data on pre-existing morbidity (chronic cardiac disease, hypertension, chronic pulmonary disease, asthma, chronic kidney disease, HIV, diabetes, current smoking, tuberculosis, malignant neoplasm), pre-admission and medications received on the day of admission or following (antiviral, corticosteroid, chloroquine/hydroxychloroquine), admission to intensive care unit (ICU) or high dependency unit (HDU), supplemental oxygen given, use of non-invasive ventilation, use of invasive ventilation, outcome (discharged alive, hospitalized, transfer to another facility, death, palliative discharge), and outcome date. Following data cleaning, data recording, and removal of missing observations we were left with 3,462 records for data analysis. Two states (Delta and Taraba) were excluded because the states each had less than 10 records: Delta (6), and Taraba (1).

### Study Variables

The primary outcome variable was death for a PCR-confirmed infection with the SARS-COV-2 virus. The secondary outcome of interest was time from enrolment to death. We coded the primary study outcome as 1 if the patient died or 0 if the outcome was censored. The time following admission was right censored at 84 days post-enrolment. The main explanatory variable was the severity of the disease. We defined the severity of disease as a composite score derived from any or all of:

1. Received supplemental oxygen therapy on any day during hospitalization,
2. Admitted to the ICU or HDU at any point during a hospital stay
3. Received non-invasive ventilation (CPAP/BIPAP) on any day during hospitalization, and
4. Received invasive ventilation (mechanical ventilator) on any day during hospitalization

Each of these variables was assigned a score of 1 if reported in the database or 0, if not reported. The severity score was calculated as the sum of these individual scores with a range of 0 to 4. The other explanatory variables were demographic (age, sex, healthcare worker status), pre-existing morbidity, malaria infection (diagnosed by microscopy or rapid diagnostic test), history of HIV infection, pre-admission and chronic medications use, and medications received on admission or following admission. These explanatory variables were coded as 1 if reported or 0 if not reported or missing.

### Statistical Analysis

We performed univariate analysis using the Fisher exact test, or the Wilcox on test as appropriate to describe characteristics of patients who had non-missing outcome data Multilevel regression modelling was used for the inferential data analysis in this study. These data were from several COVID-19 treatment sites across Nigeria (Figure 1) with clustering of patients by site. The sites in turn were nested by facility type e.g., tertiary hospital, non-tertiary hospital, and other treatment site. We fitted a mixed effects logistic regression model using maximum likelihood and the bound optimization by quadratic approximation algorithm to predict mortality with severity, age group, male gender, health worker status, chronic cardiac disease, diabetes, respiratory disease (defined as presence of any of chronic pulmonary disease, active or previous tuberculosis, and asthma), chronic kidney disease, malaria, azithromycin and chloroquine entered into the model as fixed effects. The model included site and facility type as random effects. We also did univariate survival analysis using the Kaplan-Meier method to assess the relationship between time from enrolment to death and patient characteristics. We further fitted mixed effects logistic regression models and Cox proportional hazards regression models to assess predictors of COVID-19 hospital death. Study site and facility type were included in the models as random effects while severity score, patients’ sex, age, health worker status, chronic cardiac disease, hypertension, respiratory disease (any of chronic pulmonary disease, active or previous tuberculosis, asthma), chronic kidney disease, chronic neurological disorder, HIV, diabetes, current smoking, malignant neoplasm, malaria, pre-admission and medications received on the day of admission or following admission (azithromycin, chloroquine/hydroxychloroquine) were included as fixed effects. We report the models with the smallest AIC selected by stepwise regression. All statistical analysis were conducted using R version 4.1.1.5. Mixed effects Cox regression was done using the Coxme package. A p-value< 0.05 was considered statistically significant.

## Results

### Description of Cohort

Four thousand patients with a positive SARS-CoV-2 diagnosis were hospitalized in the various treatment centers across 13 high-burden states of Nigeria between 25 February 2020 and 30^th^ August 2021. Of these, 3462 had completed outcomes and so were included in this analysis. The median age was 40 years (interquartile range [IQR], 28 years, 54 years), 2,990(60.6%) were male, and 147(4.2%) were health workers. Using the severity score 3,500 patients (86.8%) had a severity score of 0. Furthermore, 299(8.6%) had prior diabetes mellitus, 594(17.2%) had hypertension and 32(0.9%) had chronic cardiac diseases (not hypertension) of these 3462 patients, 213 of them died while on admission giving a mortality rate of 6.15%. Half of these deaths occurred within two days of admission. The mean time from admission to discharge or occurrence of outcome (death) was 12 days (standard deviation, 5.6 days). See Table I. Out of those with completed outcomes (n = 3462 patients), the median age, sex, and comorbidity profiles were significantly different between those who died and those who were discharged alive (Table 1). In addition, there is a significant difference in the presence of comorbidities (Chronic cardiac diseases, diabetes, hypertension, and chronic kidney diseases) between those who died and those who were discharged alive (Table 1).

### Predictors of Mortality

The mixed levels logistic regression model showed, that male sex (odds ratio [OR], 1.78 [95% confidence interval {CI}, 1.23–2.56]) and age group 45–65 years (OR, 3.93 [95% CI, 1.29–12.02]), age group 66–75 years (OR, 5.37 [95% CI, 1.68–17.14]), age group > 75 years (OR, 6.81 [95% CI, 2.04–22.82]), having a chronic cardiac disease (OR, 3.07 [95% CI, 1.20–7.86]), being diabetic (OR, 2.16 [95% CI, 1.41–3.31]), and having chronic kidney disease (OR, 11.01 [95% CI, 2.74–44.24]),were strongly associated with increased odds of death (Table 2). However, being a health worker (OR, 0.22 [95% CI, 0.06–0.79), having a concurrent malaria infection (OR, 0.45 [95% CI, 0.16–1.28]), use of Azithromycin for treatment (OR, 0.33 [95% CI, 0.19–0.54]), and use of Chloroquine/Hydroxychloroquine for treatment (OR, 0.07 [95% CI, 0.03–0.14]) were significantly associated with decreased odds of death (Table 2). The Cox regression model of predictors of time to death showed being aged 65–75 years (HR, 3.54 [95% CI, 1.05–11.83]), aged > 75 years (HR, 3.58 [95% CI, 1.06–12.15]), and diabetic (HR, 1.99 [95% CI, 1.41–2.80]) were associated with increased hazards of death. Treatment with CQ/HCQ (HR, 0.20 [95% CI, 1.10–0.38]) was associated with a reduced risk of death (Table II).

**Figure 3:**
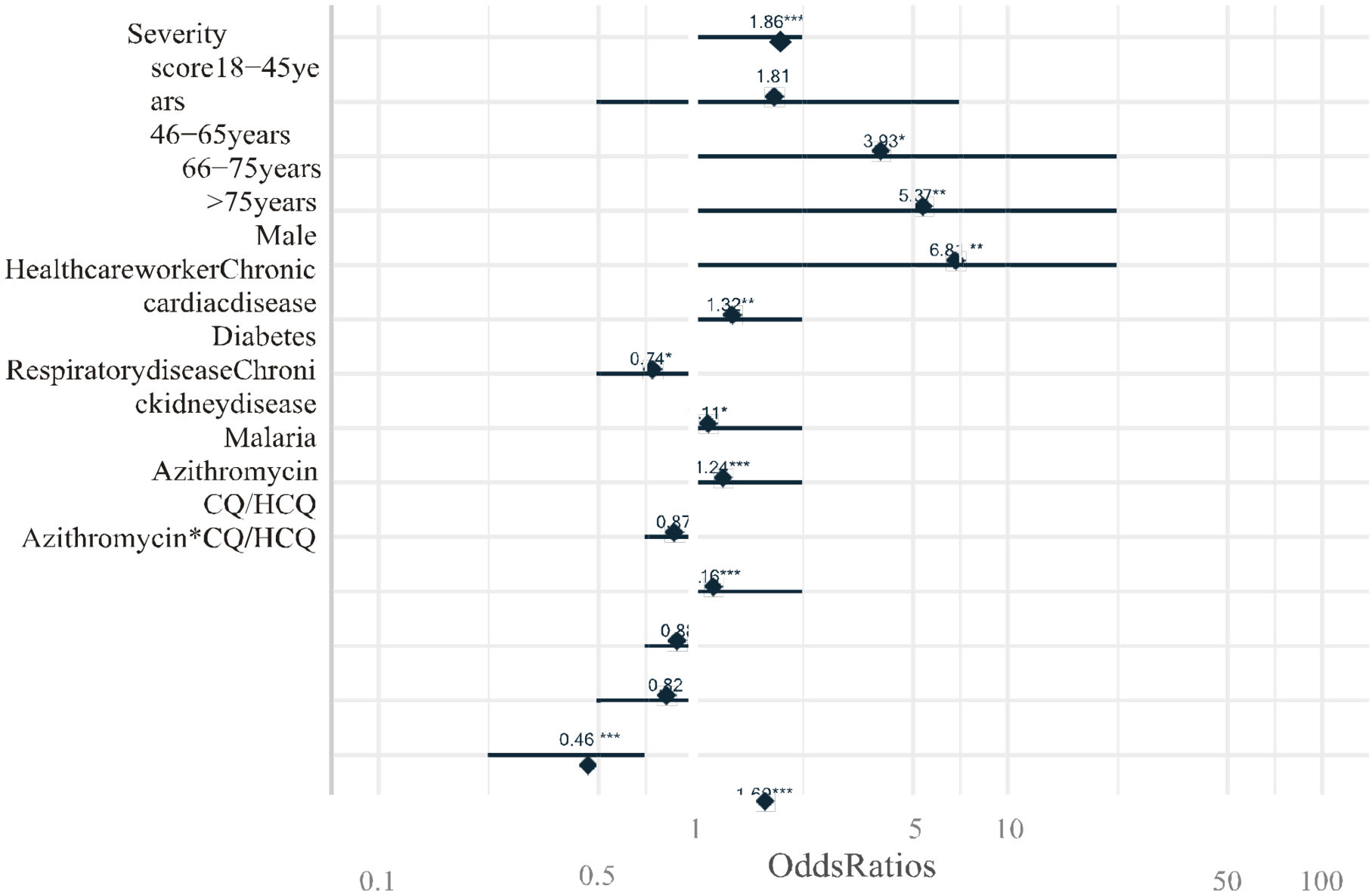
Forest plot of standardized model coefficients

The standardized model estimates using a forest plot show that age > 75 years having the largest effect on mortality while treatment with chloroquine/hydroxychloroquine has the largest protective effect (Figure 3).

#### Cumulative risk of dying (survival curves)

Using Kaplan-Meir survival analysis, the cumulative probability of death of male patients, diabetics, hypertensives, patients that were anticoagulated, patients given steroids and patients with CKD was seen to be higher than that of female patients and those without those comorbidities (Figure 4). Patients on steroids, anticoagulation and those with diabetes were dying as early as 1 week into admission 9Figure 4).

## Discussions

We present detailed information on the demographic, clinical, and outcome data for 3462 laboratory-confirmed SARS-CoV-2 cases across 13 high-burden states in Nigeria.

Overall, we recorded a higher death rate among persons admitted with COVID-19 in the isolation centers compared with many countries in sub–Saharan Africa except for South Africa which fared worse. [7,8,9] We demonstrated an increase in the death rates with an increase in the age of patients. Specifically, we found being older than 75 years to have the largest effect on the risk of death. in addition, we showed that being male, having severe disease, having chronic cardiac diseases (not hypertension), having chronic kidney disease, having respiratory disease, and being diabetic, all independently predict a higher risk of death from COVID-19.

We observed a relatively high in-hospital mortality rate in our patients similar to what has been reported in some other sub-Saharan African countries.8,9 This high death rate among hospitalized patients observed in our study may mirror the state of our healthcare delivery system.[4,5] The COVID-19 pandemic came with strain and stretch on the already weak health system where emergency care services, hospital equipment especially availability of oxygen and ventilator for those in severe respiratory failure as seen in the many admitted COVID-19 cases was lacking.4,5 Therefore, there is a need to utilized the lessons learned from these high in-hospital death rates in the current pandemic to improve on these weak points for improved healthcare delivery generally.5,7

The finding of being greater than 75 years of age is highly predictive of the risk and hazard of dying once admitted with COVID-19 among our patients in Nigeria is not unexpected as global reports have shown older persons to be disproportionately affected and also carry a higher risk of death especially the elderly.[9,10,11] The effect of aging and the associated decreased immunity to infection as well as the increased risk to respiratory pathogens in the aged and elderly have been described as potential reasons why the elderly fared worst in this COVID pandemic.[10,11] In addition, age-associated decreases in estrogen and testosterone, may mediate proinflammatory increases in older adults that could be responsible for this increase in their risk of COVID-19 adverse outcomes.[11,12,13] However, other physiological and sociodemographic factors associated with aging might have been implicated and so require more in-depth investigation in future research.

Unlike what is seen in most developed countries, our study showed people within the age bracket of 45–65 years to disproportionately carry the burden of those admitted as well as a high burden of death from COVID-19. This may not be unconnected with the low mean age and life expectancy in Nigeria.[14] This low mean population age and life expectancy leads to a diamond-shaped population demographics consisting of mainly young and middle age persons.[14,15] And since the population consists of mainly young persons, the burden and the death rates would usually fall upon the population with the largest distribution.[7,10] There is therefore the need to standardize this death rate in order to understand the true effect of age group on the mortality from COVID-19 in Nigeria.[16]

In addition, being male confers higher odds of dying like what has been reported globally. While it is not immediately clear why this is so, however, sex differences in response to inflammation as well as the role of the steroid sex hormones on immune responses have been postulated to be potentially responsible for these sex differences. [13,17] It remains to be seen if the sex difference is a true physiologic difference or rather one occasioned by chance. [13,17]

We found having chronic heart disease, being diabetic, or possessing a prior chronic kidney disease increases the odds of dying in the patients admitted with COVID-19 in our treatment facilities. In fact, having any of these comorbidities is associated with early death on admission similar to what has been reported in some earlier studies in Nigeria. [18,19] The mechanisms underlying the association between these comorbidities and COVID-19 remain to be determined. However, reports have demonstrated a possible effect of infection-related demand ischemia on the heart with subsequent myocardial injury or dysfunction as a possible causal factor.[20,21] In addition, a viral-induced inflammatory storm causing shock and ensuing ischemic-related injury, especially in a setting of a pro-inflammatory milieu such as diabetes may be a very important consideration here.[21] We however observed no significant association between hypertension and the risk of death unlike what has been reported globally. [18,20] It is probable that hypertension on its own may not be the problem but rather the complications that might have occurred in some organ systems like the heart and the kidneys as a result of long-standing hypertension.[21,22] It is a well-known fact that hypertension causes cardiac and renal dysfunction both of which were observed to increase the risk of death in our cohort.

Our study observed a protective effect of concurrent malaria infection on the risk of death in patients admitted with COVID-19. This is a significant finding, especially in light of the varied epidemiology of COVID-19 between the malaria-endemic regions of the world like sub-Saharan Africa where Nigeria is located, and the regions of the world where malaria has been virtually eliminated.[1,10,22] Some have postulated the possibility of a cross-reactive immunity to malaria as a potential protective reason accounting for the low population-level effect of the pandemic in Nigeria and many parts of sub-Saharan Africa.[21,22] More so that studies have shown a protective effect between malaria endemicity and reduced rates and risk of death from COVID-19. [25,26] There are also reports showing some cross-immunity between malaria and some viruses. [24,25] For instance, prior exposure to Plasmodium has been shown to have protective effects against Chikungunya. SARS-CoV-2 virus has been shown to use the angiotensin-converting enzyme 2 (ACE2) receptor to invade the host cells. [24,25] Similarly, studies have revealed that ACE1 and ACE2 polymorphisms protect the host from susceptibility to malaria. [28,29] In addition, there are indications that interferons generated by lymphocytes as an immune response to malaria has in vitro and in vivo efficacy against the coronavirus responsible for COVID-19. 30

We also observed that the use of chloroquine/hydroxychloroquine in the treatment of the patients admitted with COVID-19 in our facilities was significantly associated with decreased risk of death. Similarly, patients treated with azithromycin had decreased risk of death. In addition, patients treated for malaria had a reduced risk of death though that was not statistically significant. Our data did not show any difference in outcomes between those on malaria who had chloroquine and those who did not have chloroquine.[31,32] While most trials tend to show a lack of clinical usefulness or an associated high risk of death, our finding however demonstrated a significantly reduced risk of death in patients who had chloroquine/hydroxychloroquine treatment on admission.[31,32] One possible reason for the observed difference between our cohort and other studies reported may be the dose and experience of the prescribers.[33,34] While our treatment centers used a lower treatment dose (400mg daily for 5 days), most of the trial and observational studies reported a dosage of between twice to even three times the dose used in our patients.[33,34] Considering the dysrhythmic side effects of the drug, it is likely that its beneficial effect might have been blunted by side effects resulting from the high doses used.[33,34]

However, in patients where azithromycin and chloroquine/hydroxychloroquine were used concomitantly, there is a blunting effect resulting in an increased risk of death. Why this is so is not immediately clear, however considering the cardiovascular dysrhythmic side effects of both drugs, it is very possible that the effect here might be related to the side effects as adding the two drugs might have resulted in amplifying the cardiovascular dysrhythmic effects with resultant increased cardiac events leading to a higher death rate.[34,35] It is thus important that further research be done especially looking closely at the cardiovascular as well as other potential reasons for this observation.

### Strength of the study

This study draws strength from two basic considerations. First, our study is the largest Nigerian study on COVID-19 patients treated during the pandemic. It also provides methodologically robust evidence on the factors associated with increased odds of COVID-19 mortality (age, sex, severity of disease on admission, and presence of comorbidities) using a large countrywide dataset. Secondly, our dataset covers the high-burden states, distributed within the northern and southern regions of the country thus providing some geographical distribution and coverage of the entire population. It also provides insight into the relative risk of dying with COVID-19 in Nigeria, where hitherto information on COVID-19 deaths is relatively scarce.

### Limitations of the study

This review could have provided information on the relative contributions of laboratory and physiologic parameters such as blood chemistry and haematologic changes in these patients. However, because of our poor laboratory support and the nonuniform utilization of the same laboratory protocols, such information was not available and so could not be used. However, our study provides important preliminary data on some clinical and demographic factors associated with risks of COVID-related mortality in Nigeria thereby adding to the body of knowledge as well as providing the needed information for appropriate public health decisions and interventions.

### Public health significance of our findings

Our data represent a wider section of the Nigerian population covering the high-burden states accounting for the majority of the total admissions for COVID-19 in Nigeria. Therefore, our findings can be used to gauge the performance of the Nigerian health system with regard to the clinical management of the disease.5,33 In addition, the findings can help us identify the weak points within the system that may need to be addressed in order to help strengthen the emergency response to current and future pandemics.[5,36] For instance, the laboratory support which was lacking could form a need for future improvement in the laboratory component of the response.[35,36]

## Conclusions

In this study, we found that male sex, older age of the patient, the presence of comorbidities like diabetes, and chronic heart, and kidney diseases on admission were associated with increased risk of mortality due to COVID-19 infection while concurrent malaria infection and use of chloroquine/hydroxychloroquine and azithromycin were associated with a decreased risk of mortality in patients treated in our isolation centers. This information is significant in that it updates our understanding as well as provides guidance to physicians managing patients with COVID-19 and may help to improve care for patients through risk stratification. It also helps our health policymakers in reaching public health decisions on the allocation of healthcare resources and personnel in this current and future pandemic.

## Figures and Tables

**Figure 2:**
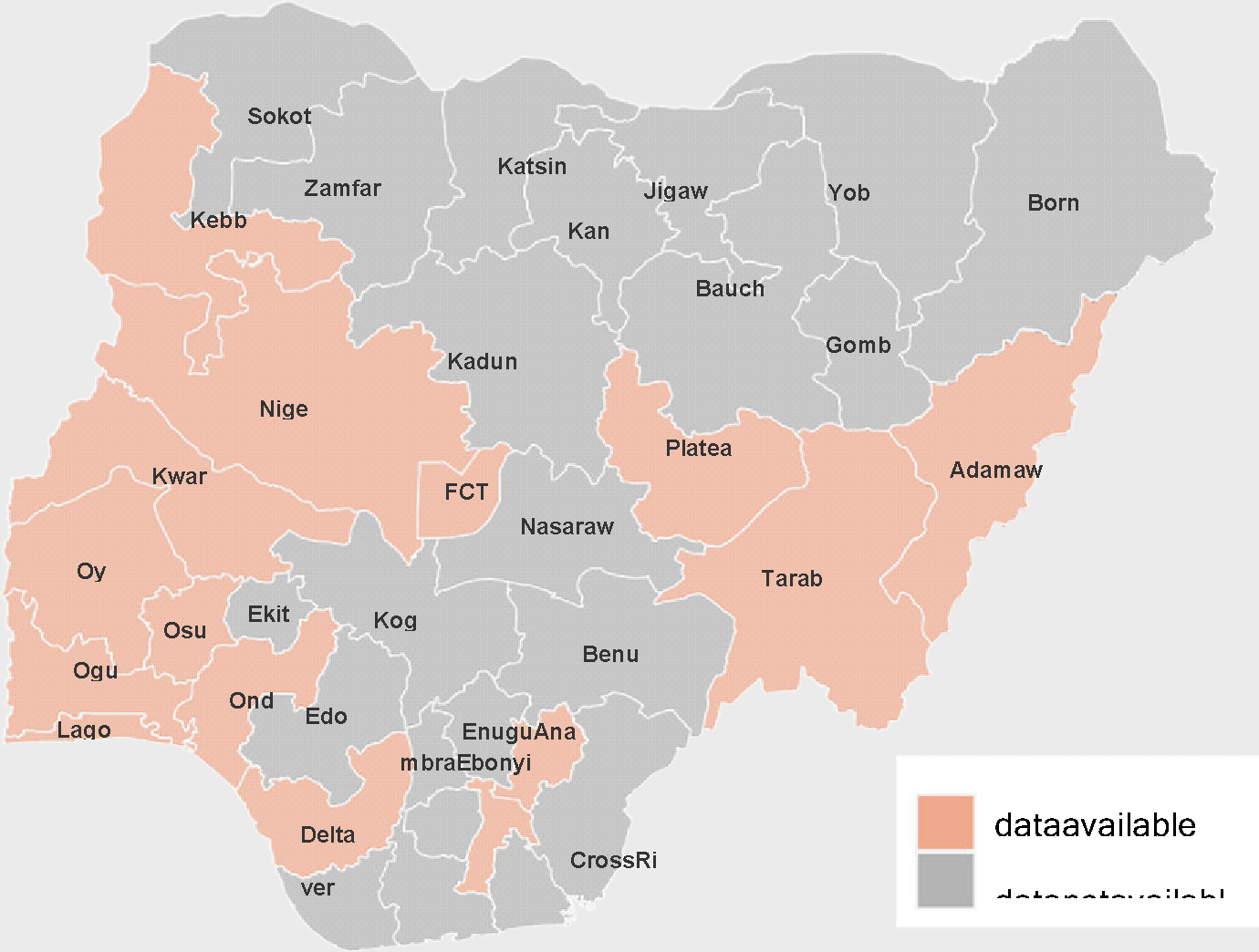
States with available data

**Figure 4a:**
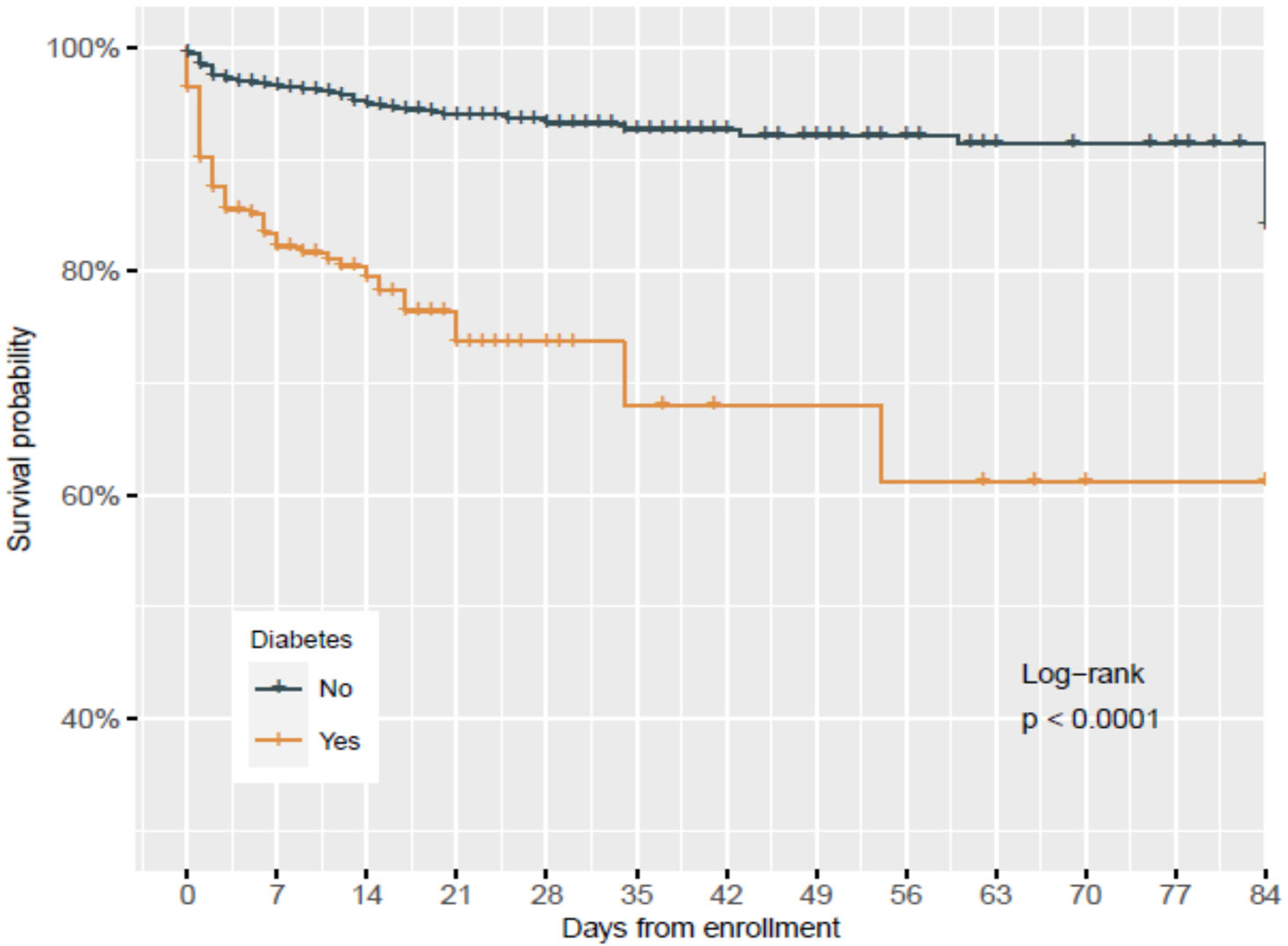
Survival curves of patients stratified by diabetes status.

**Figure 4b:**
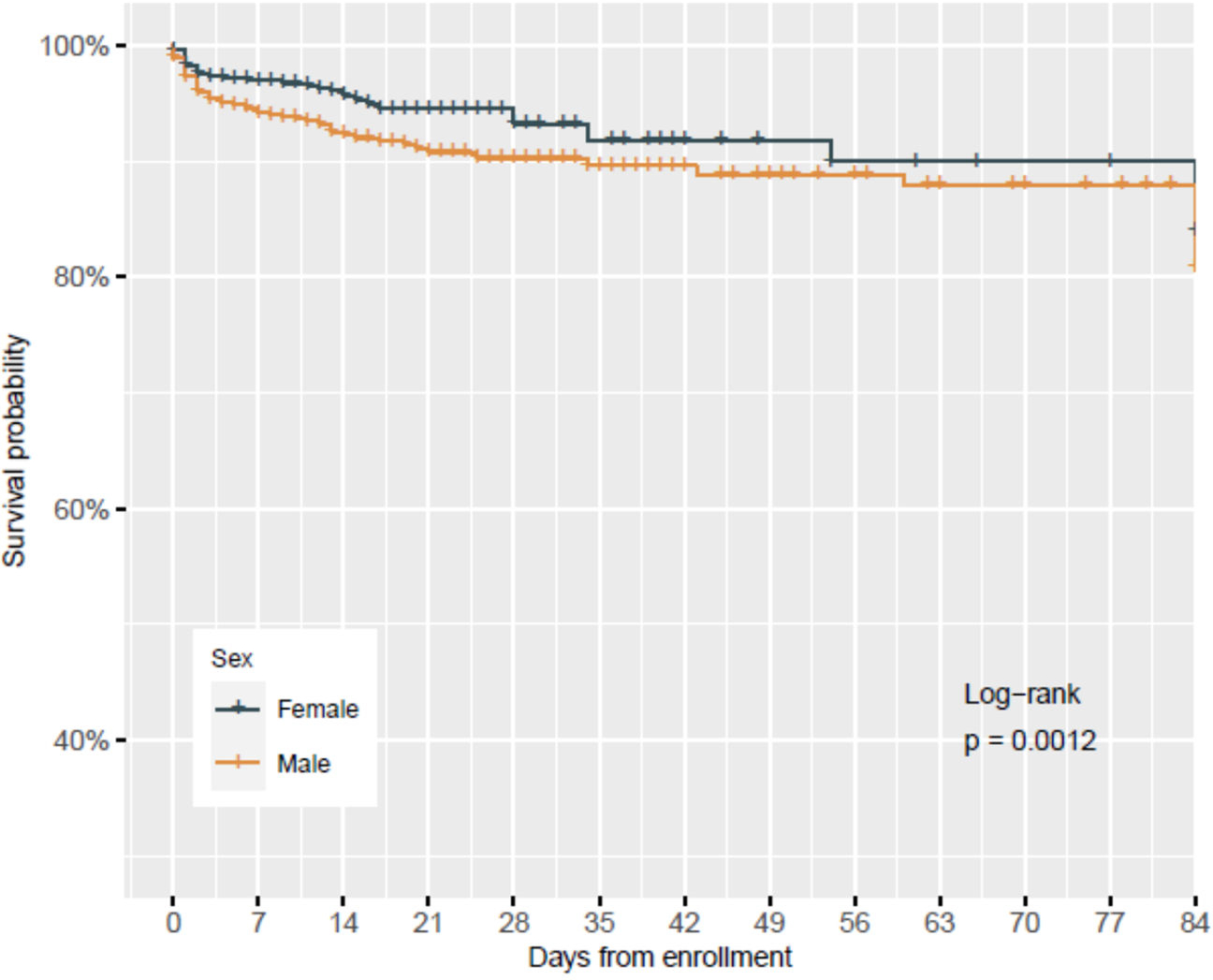
Survival curves of patients stratified by gender.

**Figure 4c:**
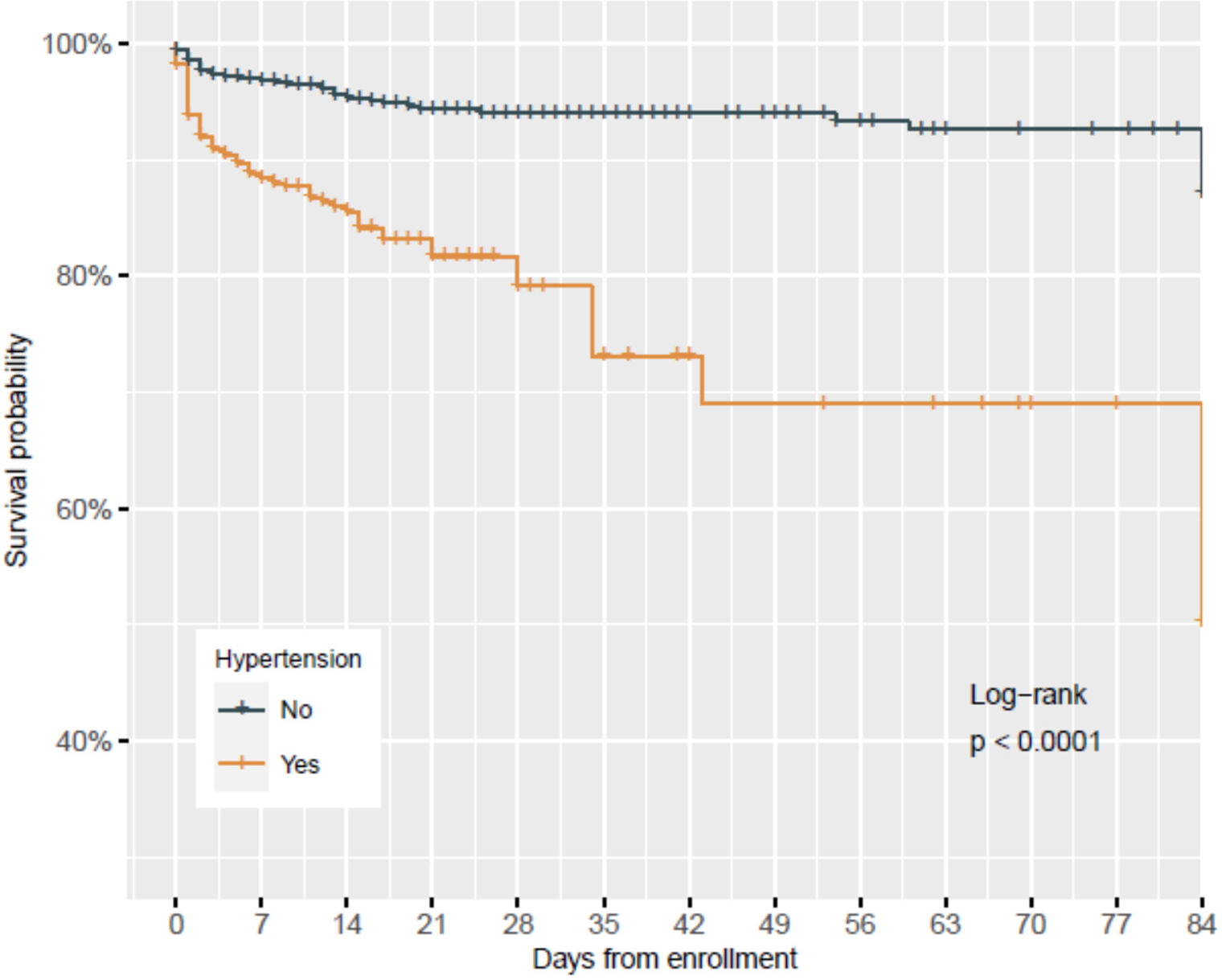
Survival curves of patients stratified by hypertension status.

**Figure 4d:**
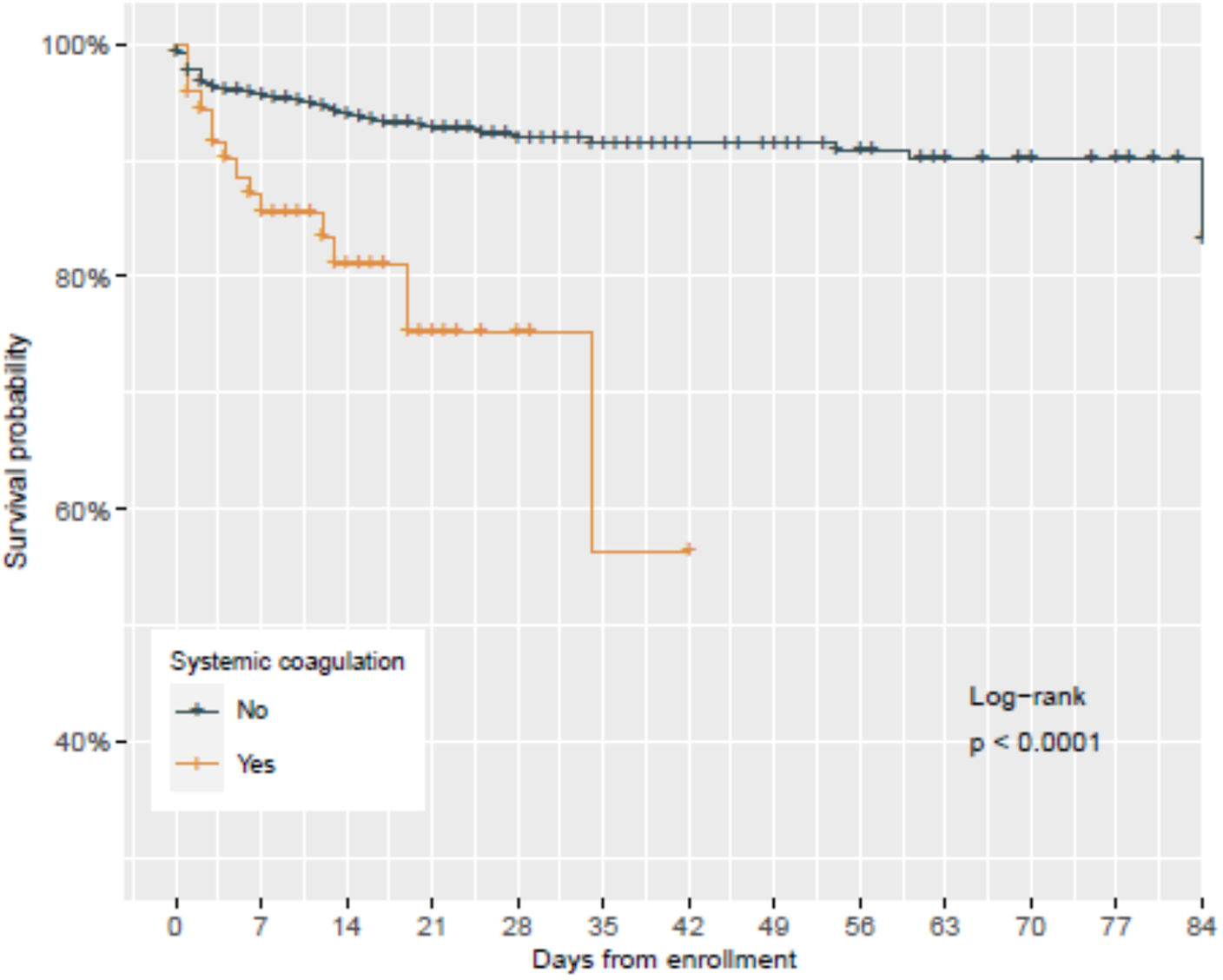
Survival curves of patients stratified by Systemic coagulation use.

**Figure 4e:**
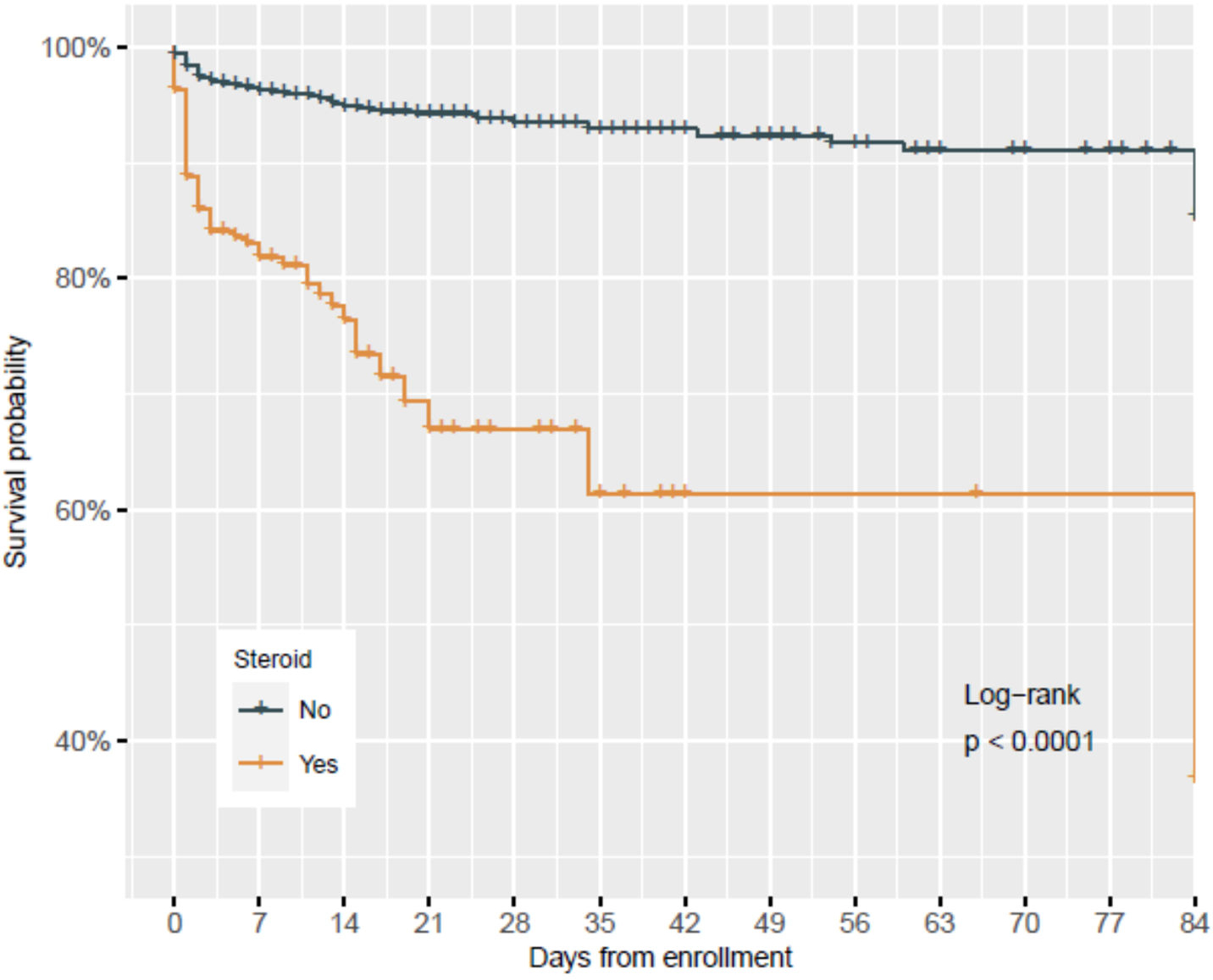
Survival curves of patients stratified by Steroid use.

**Table 1:** Patient characteristics

	Died		
Variables	No,N=3,249	Yes,N=213	pvalue

Severity score			**<0.001**
0	2,916(89.8%)	89(41.8%)	
1	249(7.7%)	71(33.3%)	
2	78(2.4%)	47 (22.1%)	
3	5(0.2%)	5(2.3%)	
4	1 (0%)	1(0.5%)	
Days from enrollment to outcome, Median (IQR)	12(7,15)	2(1,10)	**<0.001**
Age, Median (IQR)	39(28, 53)	61 (48,70)	**<0.001**
Age group			**<0.001**
<18	252(8.1%)	4(1.9%)	
18–45	1,688(54.4%)	39(18.7%)	
46–65	894(28.8%)	95(45.5%)	
66–75	183(5.9%)	41(19.6%)	
>75	87(2.8%)	30(14.4%)	
Male	1,947(59.9%)	152(71.4%)	**<0.001**
Healthcare worker	144(4.4%)	3(1.4%)	**0.034**
Chronic cardiac disease (not hypertension)	19(0.6%)	13(6.1%)	**<0.001**
Diabetes	237 (7.3%)	62(29.1%)	**<0.001**
Hypertension	506(15.6%)	88(41.3%)	**<0.001**
Current smoking	18(0.6%)	4(1.9%)	**0.042**
Respiratory disease	55(1.7%)	4(1.9%)	0.782
Chronic neurologic disease	12(0.4%)	0 (0%)	>0.999
Malignant neoplasm	8(0.2%)	2(0.9%)	0.122
Chronic kidney disease	10(0.3%)	6(2.8%)	**<0.001**
HIV	24(0.7%)	4(1.9%)	0.09
Malaria	78(2.4%)	6(2.8%)	0.702
Antiviral	457(14.1%)	24(11.3%)	0.253
ACE-i/ARB	94(2.9%)	10(4.7%)	0.136
Azithromycin	1,515(46.6%)	104(48.8%)	0.534
Corticosteroid	180(5.5%)	54(25.4%)	**<0.001**
CQ/HCQ	968(29.8%)	71(33.3%)	0.275
NSAIDs	31(1%)	3(1.4%)	0.463
Systemic anticoagulation	58(1.8%)	15(7%)	**<0.001**

ACE-i/ARB=Angiotensin-converting enzyme inhibitors; NSAIDs=non-steroidal anti-inflammatory drugs; CQ/HCQ=Chloroquine/hydroxychloroquine Fisher’s exact test; Wilcoxon rank-sum test; Pearson’s Chi-squared test

**Table 2:** Mixed effects logistic regression model of predictors of mortality

Predictors	OR	95%CI	pvalue

Severity score	3.50	2.73,4.49	<0.001
Age group			
<18	...	...	
18–45	1.81	0.58,5.61	0.307
46–65	3.93	1.29,12.02	0.016
66–75	5.37	1.68, 17.14	0.005
>75	6.81	2.04,22.81	0.002
Male	1.78	1.23,2.56	0.002
Healthcare worker	0.22	0.06,0.79	0.020
Chronic cardiac disease (not hypertension)	3.07	1.20,7.86	0.019
Diabetes	2.16	1.41,3.31	<0.001
Respiratory disease	0.34	0.09,1.20	0.094
Chronic kidney disease	11.01	2.74,44.25	<0.001
Malaria	0.45	0.16,1.28	0.134
Azithromycin	0.33	0.19,0.58	<0.001
CQ/HCQ	0.07	0.03,0.14	<0.001
Azithromycin*CQ/HCQ	9.69	4.08,23.04	<0.001

Number of observations	3313		
ConditionalR^2^	0.55		
MarginalR^2^	0.25		
Log-likelihood	−507.31		
AIC	1050.61		

OR=Odds Ratio, CI=Confidence Interval, CQ/HCQ=Chloroquine/hydroxychloroquine.
